# Embedding laboratory animal welfare- and ethics-related techniques into a comparative medicine laboratory course: an educational reform study

**DOI:** 10.3389/fvets.2026.1926120

**Published:** 2026-08-28

**Authors:** Jian Zhang, Lingyan Zhang, Xingtao Zhang, Gang Yao

**Affiliations:** 1 Institute of Laboratory Animal Science, Guizhou University of Traditional Chinese Medicine, Guiyang, China; 2 Outpatient Department, The Second Affiliated Hospital of Guizhou University of Traditional Chinese Medicine, Guiyang, China

**Keywords:** comparative medicine, embedded teaching, ethics education, laboratory animal welfare, practical teaching

## Abstract

**Background:**

Animal welfare and ethics education is essential for laboratory animal science training, but students often struggle to translate ethical principles into practical experimental procedures. This study explored an embedded teaching approach that integrated animal welfare- and ethics-related techniques into a comparative medicine laboratory course using the bilateral ovariectomy (OVX) mouse model as an authentic teaching scenario.

**Methods:**

A pre–post educational intervention design was adopted. Students first independently wrote welfare- and ethics-related technical plans for the OVX experiment. Instructors then provided structured teaching covering model justification, anesthesia and analgesia, perioperative care, postoperative monitoring, humane endpoints, sample collection, euthanasia, welfare recording, and institutional compliance. After instruction, students revised their plans using the same assessment criteria and subsequently performed OVX procedures under instructor supervision. Their technical standardization and welfare-related performance were evaluated using a predefined practical rubric. A post-course questionnaire was used to assess students’ perceptions of the teaching model.

**Results:**

After instructor explanation, students’ correct response rates improved across all assessed welfare domains, particularly in model justification, surgical procedures, analgesia, welfare recording, and intervention strategies. In the practical assessment, 80 students achieved a mean score of 81.96 ± 5.86, with a median score of 83.00; 65.0% scored 80 or above, and no student scored below 70. Post-course questionnaire results showed strong acceptance of the embedded teaching model, with all dimensional mean scores exceeding 4.40 on a 5-point Likert scale. The highest scores were observed for attitude and ethical awareness, overall evaluation, and OVX scenario-based learning effect. However, score-loss analysis and questionnaire findings revealed remaining weaknesses in anesthesia depth monitoring, analgesia implementation, intraoperative welfare protection, postoperative observation, welfare documentation, and humane endpoint-related decision-making.

**Conclusion:**

Embedding animal welfare- and ethics-related techniques into OVX-based experimental teaching was associated with improvements in students’ understanding, attitudes, and practical application of welfare-related procedures. This approach may help students progress from abstract ethical awareness to practical ethical competence and provides a feasible framework for animal welfare and ethics education in laboratory animal science programs.

## 1 Introduction

Laboratory animals play an indispensable role in biomedical research, particularly in the investigation of disease mechanisms and the development of therapeutic strategies. With the increasing complexity of experimental models and growing public concern regarding animal welfare, the ethical and humane use of laboratory animals has become a fundamental requirement for responsible scientific research (1, 2). Accordingly, laboratory animal welfare and ethics education has been widely incorporated into biomedical training programs worldwide (3).

Despite these efforts, a persistent challenge remains: students often acquire knowledge of ethical principles without developing the practical competence required to apply animal welfare considerations during real experimental procedures. Traditional animal welfare education frequently emphasizes general concepts, such as the 3Rs principle and regulatory compliance, while providing limited guidance on how these principles should be operationalized in specific experimental contexts (4, 5). As a result, welfare considerations are sometimes treated as formal requirements rather than integral components of experimental design and execution.

This gap between ethical knowledge and experimental practice is particularly evident in laboratory animal science education in China (4, 6). Although students are expected to design, conduct, and evaluate animal experiments and to undertake responsibilities related to animal welfare management and ethical review, current training approaches often emphasize theoretical knowledge and technical procedures over welfare-based decision-making. Consequently, students may be familiar with ethical principles and the 3Rs concept, yet remain insufficiently prepared to apply them in dynamic experimental contexts, including pain and distress assessment, analgesic strategy selection, procedural refinement, and humane endpoint determination. This highlights the need for an integrated teaching model that embeds animal welfare and ethics into experimental training and strengthens students’ practical ethical competence.

Comparative medicine provides a unique educational framework for addressing this challenge. By emphasizing both the similarities and differences between animal models and human diseases, comparative medicine naturally integrates scientific inquiry with ethical reflection on the use of animals in research (7, 8). Laboratory courses in comparative medicine typically include disease model construction, experimental intervention, and outcome assessment, offering authentic experimental contexts in which animal welfare issues naturally emerge. These characteristics make comparative medicine laboratory courses particularly suitable for integrating animal welfare training into experimental teaching.

In this context, embedding animal welfare techniques directly into experimental courses represents a promising approach to enhance students’ practical ethical competence. Rather than treating animal welfare as an isolated topic, an embedded teaching model integrates welfare-related technical skills-such as pain and stress assessment, analgesia planning, and humane endpoint determination-into routine experimental tasks. This approach allows students to practice ethical decision-making alongside scientific experimentation, fostering a more integrated understanding of responsible animal research.

In the present study, we selected the bilateral ovariectomy (OVX) mouse model as a representative experimental scenario to embed animal welfare and ethics training into a comparative medicine laboratory course. The OVX model involves multiple welfare-sensitive procedures, including surgical modeling, anesthesia and analgesia, perioperative care, postoperative monitoring, humane endpoint determination, and euthanasia (9). Therefore, it provides an appropriate teaching context for guiding students to translate ethical principles into operational welfare techniques. Using a pre–post protocol-based assessment and instructor evaluation during experimental practice, this study aimed to determine whether OVX model-based embedded teaching could improve students’ practical ethical competence in laboratory animal science education.

## 2 Methods

### 2.1 Study design

This study adopted a pre-post educational intervention design to evaluate the effect of embedded animal welfare and ethics training in a comparative medicine laboratory course (10). The course was offered to third-year undergraduate students as a 3-credit course. The educational intervention evaluated in this study consisted of 8 instructional hours, including 4 h of theoretical instruction and 4 h of practical OVX procedures. The teaching intervention was conducted using the bilateral ovariectomy (OVX) mouse model as the experimental scenario. The evaluation process consisted of four sequential steps: first, students independently wrote welfare- and ethics-related technical plans for the OVX experiment before formal instruction; second, instructors provided systematic teaching on animal welfare and ethical techniques involved in the OVX model; third, students revised or rewrote their welfare-related technical plans after the teaching intervention; and finally, students performed the OVX experimental procedures under instructor supervision, during which their technical standardization and welfare-related performance were evaluated. This study, including the OVX procedures involved in the teaching activity, was approved by the Ethics Committee of The Second Affiliated Hospital of Guizhou University of Traditional Chinese Medicine (LW20260530).

### 2.2 Participants and course setting

The teaching practice was implemented in the Comparative Medicine Laboratory Course for undergraduate students majoring in Laboratory Animal Science. Before participating in this course, students had completed foundational courses in laboratory animal science, biomedical theory, and basic experimental techniques. The OVX model was selected as the teaching case because it involves surgical modeling, perioperative care, postoperative monitoring, humane endpoint assessment, and sample collection, thereby providing an appropriate experimental context for integrating animal welfare and ethics training into practical teaching. All 80 students who completed the entire course were included in the analysis. The teaching team consisted of three instructors, resulting in a student-to-instructor ratio of approximately 27:1.

### 2.3 Pre-intervention assessment of OVX welfare- and ethics-related techniques

Before the welfare and ethics teaching session, students were required to independently write a welfare-related technical plan for the OVX experiment. The written plan focused on key ethical and welfare elements involved in the experimental process, including scientific justification for the OVX model, assessment of expected pain and distress, anesthesia and analgesia strategy, perioperative care, postoperative monitoring indicators, humane endpoint criteria, sample collection considerations, and euthanasia method.

Student responses were evaluated using a structured checklist (Supplementary Table 1) developed according to laboratory animal welfare principles and IACUC review requirements. Each item was judged as correct or incorrect based on whether the response was accurate, specific, and practically applicable. The correct rate was calculated as follows: correct rate = number of correctly described items/total number of assessed items × 100%. This pre-intervention assessment was used to determine students’ baseline ability to identify and apply welfare and ethics techniques in the OVX experimental design.

### 2.4 Teaching intervention on OVX-related animal welfare- and ethics-related techniques

After the pre-intervention assessment, instructors delivered a structured teaching session on welfare and ethics techniques related to the OVX model. The teaching content covered the entire experimental workflow. Special emphasis was placed on translating ethical principles into operational procedures. For example, students were guided to distinguish scientific endpoints from humane endpoints, select analgesic strategies according to the expected surgical burden, recognize behavioral and physiological indicators of pain or distress, and determine whether an animal should continue in the experiment, receive intervention, or be humanely euthanized. Case-based discussion and instructor-guided feedback were used to help students understand how welfare-related decisions are made during dynamic experimental processes.

### 2.5 Post-intervention assessment of OVX welfare- and ethics-related techniques

Following the teaching intervention, students were again required to write a welfare-related technical plan for the OVX experiment. The content and scoring criteria were consistent with those used in the pre-intervention assessment. The post-intervention correct rate was calculated using the same formula. By comparing the correct rates before and after instruction, the study evaluated whether the teaching intervention was associated with improvements in students’ understanding and application of OVX-related animal welfare techniques. In addition to the overall correct rate, improvements in specific domains were analyzed. This allowed the evaluation to identify which aspects of welfare-related competence were most effectively improved through the teaching intervention.

### 2.6 Practical assessment during OVX experimental procedures

After completing the written assessments and teaching session, students performed the OVX experimental procedures under instructor supervision. Considering the time constraints of the laboratory course, the practical teaching session focused on the key perioperative procedures, including preoperative preparation, completion of OVX surgery, and postoperative observation until recovery from anesthesia. Instructors evaluated students’ technical standardization and welfare-related performance using a predefined practical assessment rubric (Supplementary Table 2). The evaluation focused on whether students could correctly apply the welfare and ethics techniques discussed during the teaching session in a real experimental setting.

### 2.7 Post-course questionnaire evaluation

The questionnaire was evaluated using a 5-point Likert scale, with 1 indicating “strongly disagree” and 5 indicating “strongly agree” (11). The questionnaire consisted of seven sections: overall course recognition, knowledge acquisition of animal welfare and ethics, improvement in practical ethical competence, changes in attitudes and ethical awareness, recognition of embedded teaching methods, learning effect of the OVX experimental scenario, and overall evaluation (Supplementary Table 3). The score of each dimension was expressed as the mean score of its corresponding items, with higher scores indicating more positive student evaluations of that dimension.

### 2.8 Data analysis

Data from the written assessments were summarized using descriptive statistics. The correct rates of OVX-related welfare and ethics techniques before and after the teaching intervention were compared to evaluate changes in students’ knowledge application ability. Practical performance scores obtained during the OVX experiment were also summarized to assess students’ ability to translate welfare and ethics knowledge into standardized experimental behavior. Improvements in specific welfare domains were further analyzed to identify strengths and remaining weaknesses in students’ practical ethical competence.

### 2.9 Ethical considerations

This study was conducted as part of routine experimental teaching. The educational evaluation focused on students’ written protocols and practical performance and did not collect personally identifiable information. To comply with the 3Rs principle and IACUC ethical requirements, no negative control group involving deprivation of animal welfare measures was established. All animal procedures were conducted under standard teaching supervision and welfare protection. The study evaluated students’ competence through a pre-post self-controlled design and practical observation without compromising animal welfare.

## 3 Results

### 3.1 Improvement in students’ understanding of animal welfare- and ethics-related techniques across experimental stages

As shown in Table 1, students’ correct response rates improved across all animal welfare domains after instructor explanation, indicating that the integration of animal welfare- and ethics-related techniques into OVX-based experimental teaching was accompanied by improved understanding and application of welfare-related procedures. Before instructor explanation, students already showed relatively high awareness of some routine monitoring and compliance-related items, such as daily monitoring, sampling burden, euthanasia, documentation, and institutional compliance. However, lower correct response rates were observed in several key areas requiring integrated ethical reasoning and procedural judgment, including model justification, surgical procedure, analgesia, welfare recording, and intervention strategies.

**Table 1:** Operational protocols for animal welfare techniques at different experimental stages and students’ correct response rates before and after instructor explanation.

Experimental stage	Welfare domain	Operational details	Before instructor explanation (%)	After instructor explanation (%)
Model establishment (OVX surgery)	Model justification	Justify OVX model; define strain, age, and sample size; avoid unnecessary duplication	55.00	93.75
	Anesthesia	Use appropriate anesthesia; monitor respiration, reflexes, and temperature	78.75	97.5
	Analgesia	Administer analgesics before and after surgery; adjust based on pain signs	68.75	87.5
	Surgical procedure	Perform aseptic surgery; minimize incision size and operation time	61.25	100
	Perioperative care	Maintain body temperature; monitor mobility and feeding; provide hydration	77.5	100
	Humane endpoints	Define endpoints such as >15–20% weight loss, infection, or severe distress	80.00	96.25
Experimental intervention and observation	Daily monitoring	Record body weight, food/water intake	86.25	100
		Assess activity, grooming, posture, pain-related behaviors	82.50	100
		Monitor wound healing and infection signs	76.25	100
	Welfare recording	Use structured monitoring sheets and scoring systems	75.00	100
	Intervention strategy	Mild: observe; Moderate: adjust procedures; Severe: terminate experiment	73.75	100
Sample collection and euthanasia	Sampling method	Select appropriate routes; reduce tissue damage	82.50	100
	Sampling burden	Avoid excessive sampling; follow tolerable limits	83.75	100
	Humane endpoints	Continuously compare observed condition with predefined criteria	80.00	100
	Euthanasia	Use approved methods; ensure rapid unconsciousness and minimal distress	81.25	100
	Documentation	Record rationale for endpoint and euthanasia decisions	82.50	100
Whole-process supervision	Full-process management	Combine protocol design, monitoring, and endpoint evaluation	81.25	100
	Compliance	Align with IACUC and institutional guidelines	83.75	100

Data were obtained from 80 students. For each welfare domain, the percentage represents the proportion of students who correctly described the corresponding item in their written welfare-related technical plan. Each student was assessed using the same checklist before and after instructor explanation.

The most pronounced improvement was observed in model justification, where the correct response rate increased from 55.00% before explanation to 93.75% after explanation. This suggests that students initially had insufficient understanding of how to justify the OVX model, define appropriate animal characteristics and sample size, and avoid unnecessary duplication. After targeted instruction, students demonstrated a clearer understanding of how animal welfare and ethical considerations should be incorporated at the experimental design stage.

Substantial improvements were also observed in surgery-related welfare techniques. The correct response rate for surgical procedures increased from 61.25 to 100%, and that for perioperative care increased from 77.50 to 100%. These findings reflected students’ enhanced understanding of aseptic techniques, minimization of incision size and operation time, maintenance of body temperature, and postoperative observation of mobility, feeding, and hydration status.

During the experimental intervention and observation stage, all assessed items reached 100% correct response rates after instructor explanation. In particular, welfare recording and intervention strategies improved from 75.00 and 73.75%, respectively, to 100%. This result indicates that students developed a more systematic understanding of structured monitoring sheets, scoring systems, and graded intervention strategies based on the severity of animal welfare concerns.

In the sample collection and euthanasia stage, students showed relatively high baseline correct response rates, ranging from 80.00 to 83.75%, and all items increased to 100% after instructor explanation. These results suggest that students further consolidated their understanding of appropriate sampling methods, reduction of sampling burden, continuous assessment of humane endpoints, approved euthanasia methods, and documentation of endpoint and euthanasia decisions.

Overall, these findings demonstrate that students showed a higher level of understanding and application of animal welfare techniques throughout the experimental process after participating in the embedded teaching activities. The improvement was especially evident in areas involving ethical justification, surgical welfare control, welfare documentation, and decision-making based on animal condition. These results support the effectiveness of embedding animal welfare and ethics education into specific experimental teaching scenarios, with students demonstrating a transition from basic awareness of welfare requirements toward the practical consideration of welfare techniques in protocol design, experimental operation, monitoring, intervention, and endpoint management.

### 3.2 Practical assessment revealed overall proficiency and remaining weaknesses in welfare- and ethics-related OVX procedures

**Figure 1:**
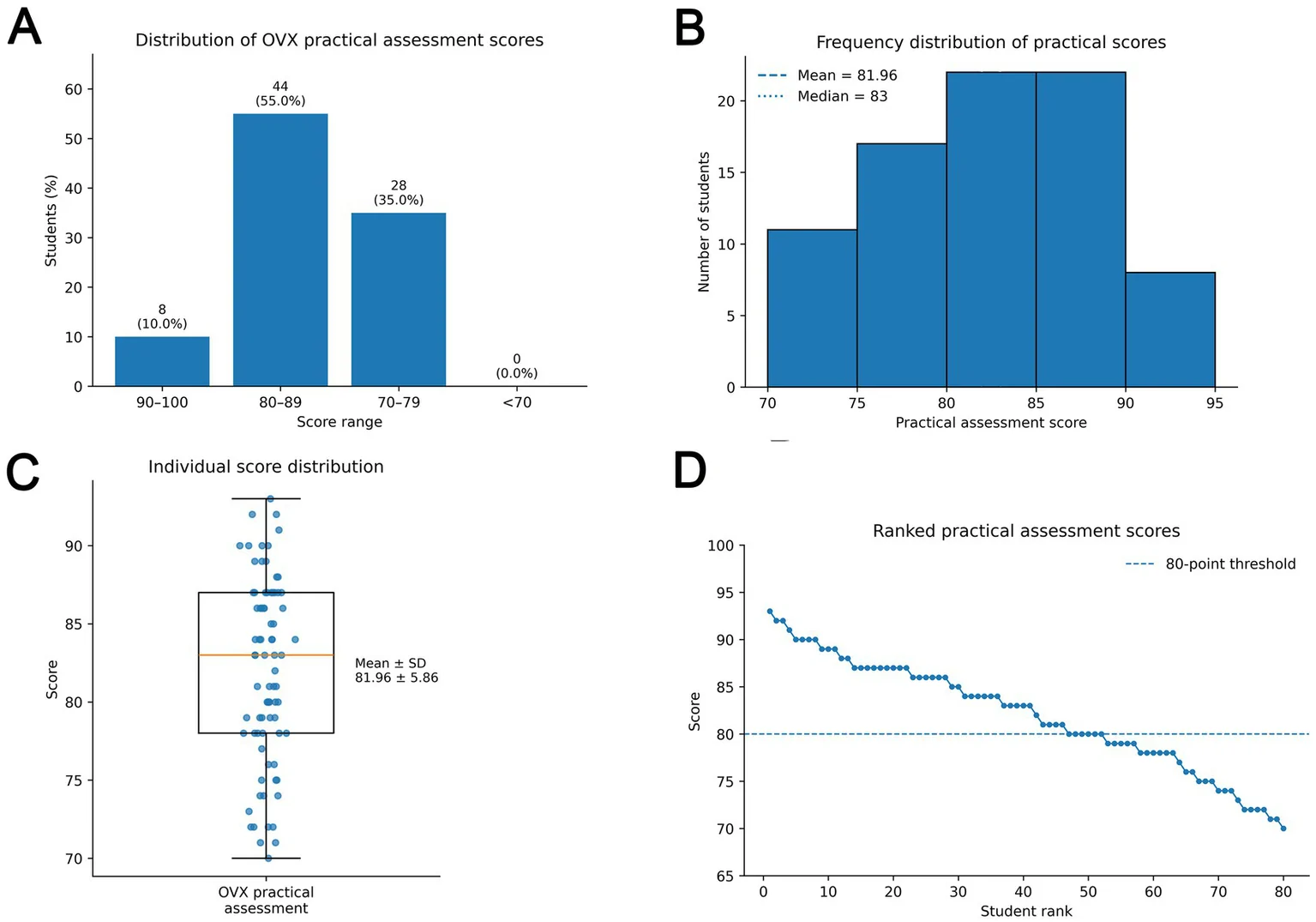
OVX practical assessment score analysis. **(A)** Distribution of OVX practical assessment scores by score range. The proportions of students scoring 90–100, 80–89, 70–79, and below 70 were 10.0, 55.0, 35.0, and 0.0%, respectively. *n* = 80. **(B)** Frequency distribution of practical assessment scores. The dashed line indicates the mean score (81.96), and the dotted line indicates the median score (83). *n* = 80. **(C)** Box plot with individual student scores. Each point represents one student. The box plot summarizes the median and interquartile range; the mean score was 81.96 ± 5.86. *n* = 80. **(D)** Ranked distribution of individual practical assessment scores. Scores were arranged in descending order to show the overall performance pattern and individual variability. *n* = 80.

After the instructor explained the animal welfare measures related to the OVX experiment, students were required to rewrite the experimental protocol according to the welfare techniques they had learned. Subsequently, OVX practical training was conducted during the laboratory session. Students performed the procedures according to their written protocols, covering the process from preoperative preparation and OVX surgery to postoperative observation until recovery from anesthesia. Using a predefined assessment rubric (Supplementary Table 2), instructors evaluated students’ practical performance in terms of technical standardization and compliance with animal welfare requirements. The assessment results are shown in Figure 1.

The mean practical score was 81.96 ± 5.86, with a median score of 83.00. The scores ranged from 70 to 93, and the interquartile range was 78–87, indicating that students’ practical performance was generally concentrated at a relatively high level. In terms of score distribution, 8 students (10.0%) achieved scores of 90 or above, 44 students (55.0%) scored between 80 and 89, and 28 students (35.0%) scored between 70 and 79. No student scored below 70. Overall, 65.0% of students achieved scores of 80 or above, suggesting that most students were able to perform OVX experimental procedures with acceptable technical standardization and animal welfare awareness.

These results indicate that the practical training may have supported students in applying animal welfare- and ethics-related techniques during OVX experimental procedures. However, the presence of a considerable proportion of students in the 70–79 score range suggests that some students still had deficiencies in procedural details and welfare-related decision-making. Regarding the potential instructor bias in practical performance assessment, we acknowledge that formal blinding was not performed. Future studies are needed to incorporate independent blinded assessors, multiple evaluators, or objective assessment approaches to enhance assessment reliability.

### 3.3 Practical assessment identified key weaknesses in welfare-sensitive OVX procedures

The practical performance assessment revealed variation among different domains of animal welfare techniques (Table 2). The highest score loss was observed in analgesia implementation (32.0%), followed by anesthesia induction and depth monitoring (24.4%) and aseptic technique (24.1%). Relatively higher score losses were also observed in intraoperative protection (21.5%) and welfare records and humane endpoint awareness (20.8%).

**Table 2:** Practical performance assessment of students in animal welfare techniques during OVX procedures (*n* = 80).

Assessment domain	Assessment indicator	Maximum score	Mean ± SD	Score loss (%)
Preoperative preparation	Animal condition evaluation	10	9.23 ± 0.56	7.70%
	Surgical instruments and environment preparation	10	9.52 ± 0.48	4.80%
Anesthesia and analgesia	Anesthesia induction and depth monitoring	10	7.56 ± 0.62	24.40%
	Analgesia implementation	10	6.80 ± 0.65	32.00%
Surgical welfare techniques	Aseptic technique	10	7.59 ± 0.60	24.10%
	Gentle animal handling	10	9.14 ± 0.60	8.60%
	Intraoperative protection	10	7.85 ± 0.63	21.50%
Postoperative care and monitoring	Postoperative warming and recovery observation	10	8.19 ± 0.61	18.10%
	Pain and distress monitoring	10	8.16 ± 0.66	18.40%
Welfare documentation and ethical decision-making	Welfare records and humane endpoint awareness	10	7.92 ± 0.64	20.80%

Three categories of items were most prone to score loss during OVX experimental procedures (Table 3). The first category was related to anesthesia and analgesia. Although most students understood that anesthesia was required for surgery, some had difficulty continuously assessing anesthesia depth based on respiratory rate, reflex responses, and body temperature. In addition, several students tended to confuse anesthesia with analgesia and paid insufficient attention to preemptive and postoperative analgesic management.

**Table 3:** Analysis of items prone to score loss during practical assessment.

Items prone to score loss	Reasons for score loss	Common problems in students’ practical operation
Anesthesia induction and depth monitoring	Anesthesia is not completed by a single drug administration; the depth of anesthesia needs to be continuously assessed.	Students often focus only on whether the animal remains immobile, while neglecting respiratory rate, pedal withdrawal reflex, corneal reflex, and body temperature. In some cases, anesthesia may be too light, causing animal movement, or too deep without timely recognition.
Analgesia implementation	Students may mistakenly regard anesthesia as equivalent to analgesia.	Common problems include remembering anesthesia but neglecting preemptive and postoperative analgesia. Students may also fail to recognize the need for additional analgesia based on pain-related signs such as hunched posture, reduced activity, wound licking, or decreased food intake.
Aseptic technique	Aseptic technique must be maintained throughout the entire surgical procedure and involves many details.	Students may perform skin disinfection and draping correctly at the beginning, but contaminate instruments, gloves, or the surgical field during the operation. For example, instruments may touch non-sterile areas and still be reused, or gloves may contact non-surgical body areas without replacement or re-disinfection.
Intraoperative protection	This item requires integrated surgical skills, including warming, tissue protection, shortening operation time, and reducing trauma.	Beginners often focus mainly on locating and removing the ovaries, while neglecting body temperature maintenance, tissue moisture, gentle traction, and incision size. Prolonged operation time, excessive tissue exposure, or rough manipulation may lead to score loss.
Postoperative warming and recovery observation	Some students may consider the procedure complete once suturing is finished.	Common problems include insufficient observation during recovery, failure to confirm respiration, activity, body temperature, and consciousness recovery, returning animals to the cage before full recovery, or providing inadequate warming support.
Pain and distress monitoring	Pain and distress signs in animals can be subtle and require practical experience to identify.	Students may only record body weight, food intake, and water intake, while overlooking abnormal posture, piloerection, reduced activity, huddling, increased aggression, wound licking, or reduced grooming behavior.
Welfare records and humane endpoint awareness	This item assesses both documentation habits and ethical decision-making ability.	Students may provide incomplete records or simply write “normal” without specific observation indicators. Their understanding of humane endpoints may remain theoretical, and they may be unable to propose appropriate intervention or termination decisions based on weight loss, infection, severe pain, or delayed recovery.

The second category involved intraoperative welfare protection. During OVX surgery, students often focused primarily on completing ovarian removal, while relatively less attention was given to welfare-sensitive technical details, including maintenance of aseptic conditions, prevention of hypothermia, minimization of incision size, reduction of tissue traction, shortening of operation time, and avoidance of unnecessary tissue trauma. These findings suggest that surgical skill training should be combined with real-time welfare supervision rather than focusing only on successful model establishment.

The third category was postoperative monitoring, welfare recording, and humane endpoint-related decision-making. Some students regarded the surgical procedure as completed after wound closure and did not sufficiently monitor recovery of consciousness, respiration, activity, feeding behavior, pain-related signs, or wound healing. Welfare records were sometimes incomplete or descriptive rather than structured, and students showed limited ability to connect abnormal animal conditions with appropriate intervention strategies or humane endpoint decisions. These results indicate that students’ practical ethical competence needs to be strengthened through whole-process training covering postoperative care, pain and distress assessment, welfare documentation, and endpoint judgment.

### 3.4 Post-course evaluation suggests the potential value of embedded animal welfare and ethics teaching

The post-course questionnaire results demonstrated strong student acceptance of the embedded animal welfare and ethics teaching model (Table 4). All dimensional mean scores exceeded 4.40 on a 5-point Likert scale. The highest scores were observed for attitude and ethical awareness (4.88), overall evaluation (4.85), and OVX scenario-based learning effect (4.83), suggesting positive student perceptions of the course in relation to ethical awareness and contextual understanding of animal welfare practices. The OVX model appeared to be particularly useful in helping students understand welfare-sensitive procedures such as postoperative monitoring, sampling, euthanasia, and confirmation of death.

**Table 4:** Dimensional scores of the post-course teaching evaluation questionnaire.

Dimension	Number of items	Total score	Mean dimension score	Percentage score
Changes in attitudes and ethical awareness	5	1,951	4.88	97.55
Overall evaluation	3	1,165	4.85	97.08
Learning effect of the OVX experimental scenario	5	1,930	4.83	96.50
Recognition of embedded teaching methods	5	1,864	4.66	93.20
Knowledge acquisition of animal welfare and ethics	5	1,846	4.62	92.30
Overall course recognition	5	1,791	4.48	89.55
Improvement in practical ethical competence	6	2,134	4.45	88.92

Data were obtained from 80 students using a 5-point Likert scale. Mean dimension scores and percentage scores were calculated based on the total score of each dimension.

Recognition of embedded teaching methods also remained high, with a mean score of 4.66, supporting the value of integrating welfare training into authentic experimental procedures rather than delivering ethics instruction as isolated theoretical content. However, the practical ethical competence dimension showed a relatively lower mean score of 4.45, especially in dynamic decision-making related to animal condition assessment and humane endpoint initiation. These findings suggest that although embedded teaching may support improvements in students’ knowledge and ethical attitudes, further scenario-based practice is needed to strengthen their practical welfare decision-making ability.

## 4 Discussion

This study explored an embedded teaching approach that integrated laboratory animal welfare- and ethics-related techniques into a comparative medicine laboratory course using the OVX mouse model as the core teaching scenario. The results showed that students’ correct response rates for OVX-related welfare techniques increased after instructor explanation, and most students achieved satisfactory scores in the subsequent practical assessment. These findings suggest that embedding welfare- and ethics-related technical training into an authentic experimental workflow may support students’ understanding of animal welfare requirements and facilitate the translation of ethical principles into practical experimental behavior.

### 4.1 Bridging the gap between ethical principles and experimental practice

A major challenge in laboratory animal welfare and ethics education is that students may understand general ethical principles but lack the ability to apply them in specific experimental procedures (4, 6). Traditional teaching of animal ethics often focuses on regulations, institutional requirements, and broad concepts such as the 3Rs, whereas students may receive limited training on how to implement these principles during real experimental operations (3, 12, 13). Additionally, students often define experimental competence primarily by technical completion, whereas animal welfare requires continuous consideration throughout the entire experimental process. Factors such as excessive tissue manipulation, inadequate thermal support, or insufficient anesthesia monitoring may substantially influence animal wellbeing despite successful completion of the surgical endpoint. As a result, animal welfare may be perceived as an external requirement rather than an integral part of experimental design and execution.

The embedded teaching strategy used in this study was intended to address this gap by linking welfare and ethics education to a concrete experimental model. The OVX model involves multiple welfare-sensitive procedures, including surgical modeling, anesthesia, analgesia, perioperative care, postoperative monitoring, humane endpoint assessment, sample collection, and euthanasia. Therefore, it provides a suitable teaching context in which students can learn how welfare principles are transformed into operational procedures. By requiring students to write welfare- and ethics-related technical plans before and after instruction, this study encouraged them to consider animal welfare from the experimental design stage rather than only during the operation itself (14, 15).

The improvement in students’ written responses after instruction indicates that systematic teaching helped them identify key welfare requirements more accurately. In particular, the marked improvement in model justification, surgical procedure, analgesia, welfare recording, and intervention strategies suggests that students gained a clearer understanding of the relationship between ethical review, experimental necessity, pain management, and animal care (16). Rather than focusing solely on technical outcomes, such as whether the surgery was successfully completed, students demonstrated greater attention to incorporating welfare considerations throughout the experimental process. These findings indicate that welfare and ethics education should not remain at the level of abstract ethical awareness but should be closely connected with experimental procedures that students can observe, perform, and evaluate.

### 4.2 Educational value of technique-oriented welfare training

The present study emphasizes technique-oriented animal welfare training. Compared with principle-oriented ethics education alone, technique-oriented training provides students with practical methods for identifying, preventing, and reducing animal pain and distress (17, 18). In the OVX teaching scenario, students were trained to evaluate animal condition before surgery, select appropriate anesthesia and analgesia strategies, maintain aseptic technique, reduce tissue trauma, monitor postoperative recovery, record welfare-related observations, and recognize humane endpoint criteria. These technical elements are essential for transforming ethical responsibility into standardized experimental practice (19, 20).

The results of the practical assessment further support the educational value of this approach. Most students achieved relatively high practical scores, indicating that they were able to apply welfare-related knowledge during OVX procedures under instructor supervision. However, the score-loss analysis also revealed that students still had weaknesses in several complex and dynamic procedures, especially anesthesia depth monitoring, analgesia implementation, intraoperative welfare protection, postoperative observation, welfare documentation, and humane endpoint-related decision-making (21). These items require not only theoretical understanding but also continuous observation, procedural judgment, and real-time decision-making (22). Therefore, they represent key areas that should receive greater attention in future practical teaching.

An important implication is that students should be trained to recognize that successful model establishment is not the only goal of animal experiments. In surgical teaching, students may focus primarily on completing ovarian removal, while insufficient attention may be paid to maintaining body temperature, minimizing incision size, reducing tissue traction, shortening operation time, and observing postoperative recovery. Embedding welfare assessment into the practical scoring rubric helped shift the focus from “whether the operation was completed” to “whether the operation was completed in a standardized and welfare-compliant manner.” this change is important for cultivating responsible experimental habits (23).

### 4.3 Implications for comparative medicine and laboratory animal science education

For students majoring in laboratory animal science, the ability to integrate animal welfare into experimental planning and operation is a core professional competence. These students may later participate in animal experiment design, animal facility management, welfare monitoring, or ethical review. Therefore, teaching should not only introduce ethical concepts but also cultivate the ability to make welfare-related decisions in real experimental contexts (24).

The comparative medicine laboratory course provides an appropriate platform for this type of training. Comparative medicine emphasizes the relationship between animal models and human diseases, requiring students to understand both the scientific value and the ethical burden of disease modeling. In this context, the OVX model allows students to discuss why a model is scientifically justified, what degree of animal burden is expected, how pain and distress should be controlled, and when intervention or humane euthanasia is required. This helps students understand that animal welfare and scientific quality are not separate goals. Instead, appropriate welfare management can reduce stress-related interference, improve experimental reliability, and support more responsible use of animals (25, 26).

The teaching model described in this study is also consistent with the competence-based direction of laboratory animal education. Competence in animal experimentation includes knowledge, technical skills, ethical awareness, and decision-making ability. By combining written protocol design, instructor explanation, practical operation, and rubric-based assessment, this study established a relatively complete evaluation chain from knowledge acquisition to practical application (27). This model may be useful for other experimental courses that involve welfare-sensitive procedures, such as surgical modeling, drug administration, behavioral testing, and sample collection.

### 4.4 Limitations and future directions

Several limitations should be acknowledged. First, this study was conducted within a single course and involved students from one academic context. Therefore, the generalizability of the findings needs to be further verified in different institutions, courses, and student populations. In addition, the absence of a contemporaneous control group or historical comparison group represents another limitation. Although the single-group pre–post design allowed assessment of changes in students’ welfare-related knowledge and practical performance following the embedded teaching approach, it cannot completely exclude the influence of other factors, such as instructor guidance, course progression, or repeated exposure to experimental concepts (28). Future studies could incorporate comparative designs, including comparisons with previous cohorts receiving conventional ethics education, parallel laboratory modules without embedded welfare components, or staggered implementation across multiple classes, to strengthen causal inference regarding the educational value of integrating welfare education into experimental training (29).

Second, the assessment framework used in this study has several limitations. The structured checklist, practical assessment rubric, and questionnaire were designed based on course objectives and laboratory animal welfare principles to evaluate students’ understanding and application of essential welfare requirements. However, formal psychometric evaluation, including pilot testing, content validity assessment, internal consistency analysis, and reliability/validity testing, was not performed (30). Moreover, several checklist items reached 100% correct response rates after instruction, suggesting a potential ceiling effect. These items mainly reflected fundamental welfare requirements, such as monitoring, documentation, and compliance awareness, and may have been more sensitive for assessing foundational knowledge than for distinguishing advanced practical competence. Future studies should further validate these assessment tools using larger and independent cohorts and incorporate more comprehensive competency-based evaluation methods, such as objective structured practical examinations (OSPE), scenario-based decision-making tasks, and multi-rater assessments (31).

Third, practical performance evaluation was mainly conducted by instructors, and formal blinding of assessors was not implemented. Therefore, the possibility of observer bias cannot be completely excluded. Although structured evaluation criteria were applied to improve consistency, future studies should consider incorporating independent blinded assessors, multiple evaluators, or external experts, including veterinarians and IACUC members, to enhance the objectivity and professional relevance of practical competency assessment (32).

Fourth, the practical teaching component was limited by the duration of the course. The classroom practice mainly covered preoperative preparation, OVX surgery, and postoperative observation until recovery from anesthesia. Longer-term postoperative welfare management, humane endpoint determination, and sample collection procedures were primarily assessed through written plans and teaching discussions rather than complete longitudinal practice. Therefore, this study did not directly evaluate students’ ability to independently implement long-term animal welfare management during extended experimental periods (33). Future teaching designs should incorporate follow-up observation records, simulated humane endpoint scenarios, digital welfare scoring systems, and case-based decision-making exercises to further strengthen students’ whole-process welfare management abilities (34).

Fifth, the questionnaire results should be interpreted cautiously because self-reported perceptions may not fully reflect actual learning outcomes and may be influenced by response bias. Future studies should combine subjective evaluations with objective performance indicators and longitudinal follow-up assessments to better determine whether welfare-related competencies are retained and transferred into subsequent research training or professional practice (35).

Finally, establishing sustainable animal welfare competence requires continuous reinforcement rather than a single instructional intervention. A single course session is unlikely to fully establish long-term welfare awareness and practical proficiency (36). Therefore, animal welfare and ethics education should be considered an integrated component of laboratory animal-related curricula, with welfare principles and practical techniques embedded across different experimental courses. Repeated exposure and application in diverse experimental contexts may facilitate the gradual development of students’ professional attitudes, technical skills, and welfare-related decision-making abilities. Achieving this goal requires coordinated efforts and collaboration among all instructors involved in laboratory animal education (37).

## 5 Conclusion

Overall, this study demonstrates that embedding laboratory animal welfare- and ethics-related techniques into comparative medicine experimental teaching is feasible and educationally meaningful. Using the OVX model as an authentic teaching scenario, students showed improved understanding of welfare-related technical requirements and greater ability to apply these requirements during practical procedures following the intervention. The findings highlight the value of integrating ethical principles with concrete experimental procedures, thereby helping students move from basic ethical awareness toward practical ethical competence. This teaching approach may contribute to the cultivation of responsible, skilled, and welfare-conscious professionals in laboratory animal science.

## Data Availability

The original contributions presented in the study are included in the article/Supplementary material, further inquiries can be directed to the corresponding authors.

## References

[1] Kiani AK Pheby D Henehan G Brown R Sieving P Sykora P . Ethical considerations regarding animal experimentation. J Prev Med Hyg. (2022) 63:E255–66. doi: 10.15167/2421-4248/jpmh2022.63.2S3.2768, 36479489 PMC9710398

[2] Ferdowsian HR Beck N. Ethical and scientific considerations regarding animal testing and research. PLoS One. (2011) 6:e24059. doi: 10.1371/journal.pone.0024059, 21915280 PMC3168484

[3] Franco NH Kerton A Lewis DI. Education in laboratory animal science and the 3Rs. Lab Anim. (2023) 57:109–11. doi: 10.1177/00236772231162166, 36912087

[4] Zhang J Zhang L. Building a sustainable framework for laboratory animal welfare and ethics education: challenges and reform strategies. Front Vet Sci. (2025) 12:1684284. doi: 10.3389/fvets.2025.1684284, 41487480 PMC12756147

[5] Franco NH Sandøe P Olsson IAS. Researchers’ attitudes to the 3Rs: an upturned hierarchy? PLoS One. (2018) 13:e0200895. doi: 10.1371/journal.pone.0200895, 30110335 PMC6093608

[6] Zhang W Xie Z Fang X Wang Z Li Z Shi Y . Laboratory animal ethics education improves medical students’ awareness of laboratory animal ethics. BMC Med Educ. (2024) 24:709. doi: 10.1186/s12909-024-05703-9, 38951842 PMC11218205

[7] Macy J Horvath TL. Comparative medicine: an inclusive crossover discipline. Yale J Biol Med. (2017) 90:493–8. 28955187 PMC5612191

[8] Mobasheri A. Comparative medicine in the twenty-first century: where are we now and where do we go from here? Front Vet Sci. (2015) 2:2. doi: 10.3389/fvets.2015.00002, 26664931 PMC4672175

[9] Sophocleous A Idris AI. Rodent models of osteoporosis. Bonekey Rep. (2014) 3:614. doi: 10.1038/bonekey.2014.109 25852854 PMC4388108

[10] Alessandri G Zuffianò A Perinelli E. Evaluating intervention programs with a pretest-posttest design: a structural equation modeling approach. Front Psychol. (2017) 8:223. doi: 10.3389/fpsyg.2017.00223, 28303110 PMC5332425

[11] Sullivan GM Artino AR Jr. Analyzing and interpreting data from likert-type scales. J Grad Med Educ. (2013) 5:541–2. doi: 10.4300/JGME-5-4-18, 24454995 PMC3886444

[12] Humpenöder M Corte GM Pfützner M Wiegard M Merle R Hohlbaum K . Alternatives in education: evaluation of rat simulators in laboratory animal training courses from participants’ perspective. Animals. (2021) 11:3462. doi: 10.3390/ani11123462, 34944238 PMC8698197

[13] Hubrecht RC Carter E. The 3Rs and humane experimental technique: implementing change. Animals (Basel). (2019) 9:754. doi: 10.3390/ani9100754, 31575048 PMC6826930

[14] Smith AJ Clutton RE Lilley E Hansen KEA Brattelid T. PREPARE: guidelines for planning animal research and testing. Lab Anim. (2018) 52:135–41. doi: 10.1177/0023677217724823, 28771074 PMC5862319

[15] Percie du Sert N Hurst V Ahluwalia A Alam S Avey MT Baker M . The ARRIVE guidelines 2.0: updated guidelines for reporting animal research. PLoS Biol. (2020) 18:e3000410. doi: 10.1371/journal.pbio.3000410, 32663219 PMC7360023

[16] Díaz L Zambrano E Flores ME Contreras M Crispín JC Alemán G . Ethical considerations in animal research: the principle of 3R’s. Rev Investig Clin. (2021) 73:199–209. doi: 10.24875/RIC.20000380, 33090120

[17] Carbone L Austin J. Pain and laboratory animals: publication practices for better data reproducibility and better animal welfare. PLoS One. (2016) 11:e0155001. doi: 10.1371/journal.pone.0155001, 27171143 PMC4865140

[18] Roughan JV Flecknell PA. Evaluation of a short duration behaviour-based post-operative pain scoring system in rats. Eur J Pain. (2003) 7:397–406. doi: 10.1016/S1090-3801(02)00140-4, 12935791

[19] Richardson CA Flecknell PA. Anaesthesia and post-operative analgesia following experimental surgery in laboratory rodents: are we making progress? Altern Lab Anim. (2005) 33:119–27. doi: 10.1177/026119290503300207, 16180987

[20] Turner PV Brabb T Pekow C Vasbinder MA. Administration of substances to laboratory animals: routes of administration and factors to consider. J Am Assoc Lab Anim Sci. (2011) 50:600–13. 22330705 PMC3189662

[21] Ullman-Culleré MH Foltz CJ. Body condition scoring: a rapid and accurate method for assessing health status in mice. Lab Anim Sci. (1999) 49:319–23. 10403450

[22] Hunt JA Rogers-Scarlett S Schmidt P Thompson RR Gilley A Devine E . Validation of a rubric used for skills-based assessment of veterinary students performing simulated ovariohysterectomy on a model. J Vet Med Educ. (2023) 50:327–36. doi: 10.3138/jvme-2022-0011, 35617609

[23] Jones-Bolin S. Guidelines for the care and use of laboratory animals in biomedical research. Curr Protoc Pharmacol. (2012) 59:Appendix 4B. doi: 10.1002/0471141755.pha04bs59, 23258596

[24] Guillén J. FELASA guidelines and recommendations. J Am Assoc Lab Anim Sci. (2012) 51:311–21. 22776188 PMC3358979

[25] Poole T. Happy animals make good science. Lab Anim. (1997) 31:116–24. doi: 10.1258/002367797780600198, 9175008

[26] Meijer MK Sommer R Spruijt BM van Zutphen LFM Baumans V. Influence of environmental enrichment and handling on the acute stress response in individually housed mice. Lab Anim. (2007) 41:161–73. doi: 10.1258/002367707780378168, 17430616

[27] Tiebosch I Whitfield L Favier R. Introducing entrustable professional activities and competency-based development into workplace-based training within laboratory animal sciences. Lab Anim. (2025) 59:645–51. doi: 10.1177/00236772251351101, 40930723

[28] Spurlock DR Jr. The single-group, pre- and posttest design in nursing education research: it's time to move on. J Nurs Educ. (2018) 57:69–71. doi: 10.3928/01484834-20180123-02, 29384566

[29] Ma T Lee Y. Overview of quantitative research. Fam Med. (2026) 58:81–7. doi: 10.22454/FamMed.2026.406133, 42102714 PMC12969534

[30] Cook DA Brydges R Ginsburg S Hatala R. A contemporary approach to validity arguments: a practical guide to Kane's framework. Med Educ. (2015) 49:560–75. doi: 10.1111/medu.12678, 25989405

[31] Khan KZ Ramachandran S Gaunt K Pushkar P. The objective structured clinical examination (OSCE): AMEE guide no. 81. Part I: an historical and theoretical perspective. Med Teach. (2013) 35:e1437–46. doi: 10.3109/0142159X.2013.818634, 23968323

[32] Bok HGJ de Jong LH O'Neill T Maxey C Hecker KG. Validity evidence for programmatic assessment in competency-based education. Perspect Med Educ. (2018) 7:362–72. doi: 10.1007/s40037-018-0481-2, 30430439 PMC6283777

[33] Cate OT Carraccio C. Envisioning a true continuum of competency-based medical education, training, and practice. Acad Med. (2019) 94:1283–8. doi: 10.1097/ACM.0000000000002687, 31460916

[34] Tremblay ML Leppink J Leclerc G Rethans JJ Dolmans DHJM. Simulation-based education for novices: complex learning tasks promote reflective practice. Med Educ. (2019) 53:380–9. doi: 10.1111/medu.13748, 30443970

[35] Sargeant J Armson H Chesluk B Dornan T Eva K Holmboe E . The processes and dimensions of informed self-assessment: a conceptual model. Acad Med. (2010) 85:1212–20. doi: 10.1097/ACM.0b013e3181d85a4e, 20375832

[36] arraccio C Englander R Gilhooly J Mink R Hofkosh D Barone MA . Building a framework of entrustable professional activities, supported by competencies and milestones, to bridge the educational continuum. Acad Med. (2017) 92:324–30. doi: 10.1097/ACM.0000000000001141 26959225

[37] Van Melle E Frank JR Holmboe ES Dagnone D Stockley D Sherbino J. International competency-based medical education collaborators. A core components framework for evaluating implementation of competency-based medical education programs. Acad Med. (2019) 94:1002–9. doi: 10.1097/ACM.0000000000002743, 30973365

