# Supplementary material for: Embedding laboratory animal welfare- and ethics-related techniques into a comparative medicine laboratory course: an educational reform study
Source: Front Vet Sci. 2026 Aug 28;13:1926120. doi: 10.3389/fvets.2026.1926120 (PMC13561924; doi:10.3389/fvets.2026.1926120)
Supplement: Supplementary file 1 [file Table_1.docx]

**Supplemental materials**

**Table 1. Structured Checklist on operational protocols for animal welfare techniques at different experimental stages.**

| **Experimental stage** | **Welfare domain** | **Measures** | **Operational details** |
| --- | --- | --- | --- |
| **Model establishment (OVX surgery)** | Model justification | Justify OVX model; define strain, age, and sample size; avoid unnecessary duplication |  |
|  | Anesthesia | Use appropriate anesthesia; monitor respiration, reflexes, and temperature |  |
|  | Analgesia | Administer analgesics before and after surgery; adjust based on pain signs |  |
|  | Surgical procedure | Perform aseptic surgery; minimize incision size and operation time |  |
|  | Perioperative care | Maintain body temperature; monitor mobility and feeding; provide hydration |  |
|  | Humane endpoints | Define endpoints such as >15–20% weight loss, infection, or severe distress |  |
| **Experimental intervention and observation** | Daily monitoring | Record body weight, food/water intake |  |
|  |  | Assess activity, grooming, posture, pain-related behaviors |  |
|  |  | Monitor wound healing and infection signs |  |
|  | Welfare recording | Use structured monitoring sheets and scoring systems |  |
|  | Intervention strategy | Mild: observe; Moderate: adjust procedures; Severe: terminate experiment |  |
| **Sample collection and euthanasia** | Sampling method | Select appropriate routes; reduce tissue damage |  |
|  | Sampling burden | Avoid excessive sampling; follow tolerable limits |  |
|  | Humane endpoints | Continuously compare observed condition with predefined criteria |  |
|  | Euthanasia | Use approved methods; ensure rapid unconsciousness and minimal distress |  |
|  | Documentation | Record rationale for endpoint and euthanasia decisions |  |
| **Whole-process supervision** | Full-process management | Combine protocol design, monitoring, and endpoint evaluation |  |
|  | Compliance | Align with IACUC and institutional guidelines |  |

**Table 2. Practical assessment rubric for welfare-related performance during OVX experimental procedures**

| **Assessment domain** | **Assessment indicator** | **Evaluation criteria** | **Score** |
| --- | --- | --- | --- |
| Preoperative preparation | Animal condition evaluation | Evaluates general condition, body weight, activity, and signs of illness or distress before surgery | 10 |
|  | Surgical instruments and environment preparation | Prepares sterilized instruments, clean surgical area, heating pad, monitoring materials, and required drugs before operation | 10 |
| Anesthesia and analgesia | Anesthesia induction and depth monitoring | Selects appropriate anesthesia, performs induction correctly, and monitors respiratory rate, reflexes, and body temperature | 10 |
|  | Analgesia implementation | Applies perioperative analgesia according to the protocol and recognizes the need for additional analgesic intervention based on pain signs | 10 |
| Surgical welfare techniques | Aseptic technique | Maintains aseptic operation, avoids contamination, and follows standard surgical procedures | 10 |
|  | Gentle animal handling | Handles animals gently, minimizes restraint stress, and avoids unnecessary stimulation or injury | 10 |
|  | Intraoperative protection | Maintains body temperature, prevents drying of tissues, minimizes incision size and operation time, and reduces tissue trauma | 10 |
| Postoperative care and monitoring | Postoperative warming and recovery observation | Provides warming support and continuously observes recovery of consciousness, mobility, respiration, and feeding behavior | 10 |
|  | Pain and distress monitoring | Identifies abnormal posture, reduced activity, poor grooming, wound problems and other pain- or distress-related signs | 10 |
| Welfare documentation and ethical decision-making | Welfare records and humane endpoint awareness | Completes welfare monitoring records accurately and understands humane endpoint criteria and appropriate intervention or termination decisions | 10 |
| **Total** |  |  | **100** |

**Table 3. Questionnaire on the Outcomes of Embedded Teaching in Laboratory Animal Welfare and Ethics**

**Dimension 1: Overall Course Acceptance**

| **Item No.** | **Item** | **1** | **2** | **3** | **4** | **5** |
| --- | --- | --- | --- | --- | --- | --- |
| **A1** | Overall, this course met my learning needs. | □ | □ | □ | □ | □ |
| **A2** | The course content was highly aligned with the training objectives of the Laboratory Animal Science major. | □ | □ | □ | □ | □ |
| **A3** | The approach of embedding animal welfare and ethics content into the Comparative Medicine laboratory course was appropriate. | □ | □ | □ | □ | □ |
| **A4** | The experimental stages were connected naturally, which helped me understand the whole process of animal experimentation. | □ | □ | □ | □ | □ |
| **A5** | I believe this teaching model is suitable for promotion in other animal experimentation courses. | □ | □ | □ | □ | □ |

**Dimension 2: Mastery of Animal Welfare and Ethics Knowledge**

| **Item No.** | **Item** | **1** | **2** | **3** | **4** | **5** |
| --- | --- | --- | --- | --- | --- | --- |
| **B1** | I understand the 3Rs principles and their application in animal experiments. | □ | □ | □ | □ | □ |
| **B2** | I understand the relationship between animal welfare and experimental scientific validity. | □ | □ | □ | □ | □ |
| **B3** | I am familiar with the basic requirements of IACUC or animal experimentation ethics review. | □ | □ | □ | □ | □ |
| **B4** | I understand the difference between humane endpoints and scientific endpoints. | □ | □ | □ | □ | □ |
| **B5** | I understand the ethical basis for measures such as anesthesia, analgesia, and euthanasia. | □ | □ | □ | □ | □ |

**Dimension 3: Improvement in Practical Ethical Competence**

| **Item No.** | **Item** | **1** | **2** | **3** | **4** | **5** |
| --- | --- | --- | --- | --- | --- | --- |
| **C1** | I am able to specify animal welfare-related content in an experimental protocol. | □ | □ | □ | □ | □ |
| **C2** | I am able to assess the potential pain and stress that animals may experience according to the characteristics of the experimental model. | □ | □ | □ | □ | □ |
| **C3** | I am able to preliminarily develop plans for anesthesia, analgesia, and perioperative care. | □ | □ | □ | □ | □ |
| **C4** | I am able to identify animal discomfort based on indicators such as body weight, activity status, grooming behavior, and wound recovery. | □ | □ | □ | □ | □ |
| **C5** | I am able to make judgments on continued observation, enhanced care, or initiation of humane endpoints according to the animal's condition. | □ | □ | □ | □ | □ |
| **C6** | I am able to standardly record welfare-related measures and their rationale in experimental reports. | □ | □ | □ | □ | □ |

**Dimension 4: Changes in Attitudes and Ethical Perspectives**

| **Item No.** | **Item** | **1** | **2** | **3** | **4** | **5** |
| --- | --- | --- | --- | --- | --- | --- |
| **D1** | Compared with before the course, I place greater importance on the role of animal welfare in experimental design. | □ | □ | □ | □ | □ |
| **D2** | I more strongly agree that animal welfare is an important component of experimental scientific validity, rather than merely a formal requirement. | □ | □ | □ | □ | □ |
| **D3** | I have a better understanding of the importance of welfare management throughout the entire experimental process. | □ | □ | □ | □ | □ |
| **D4** | I am willing to actively implement welfare-related measures in future animal experiments. | □ | □ | □ | □ | □ |
| **D5** | This course enhanced my awareness of research ethics and professional responsibility. | □ | □ | □ | □ | □ |

**Dimension 5: Recognition of the Embedded Teaching Method**

| **Item No.** | **Item** | **1** | **2** | **3** | **4** | **5** |
| --- | --- | --- | --- | --- | --- | --- |
| **E1** | Case-based teaching helped me understand animal welfare and ethics issues. | □ | □ | □ | □ | □ |
| **E2** | Pre-class experimental protocol design helped me actively think about welfare issues. | □ | □ | □ | □ | □ |
| **E3** | Instructor feedback based on ethics review standards helped me improve my experimental protocol. | □ | □ | □ | □ | □ |
| **E4** | Scenario-based problem discussions helped improve my ability to make ethical decisions in experiments. | □ | □ | □ | □ | □ |
| **E5** | Embedding welfare techniques throughout the entire experimental process was more helpful for learning than teaching ethical theories separately. | □ | □ | □ | □ | □ |

**Dimension 6: Learning Effectiveness in the OVX Experimental Context**

| **Item No.** | **Item** | **1** | **2** | **3** | **4** | **5** |
| --- | --- | --- | --- | --- | --- | --- |
| **F1** | The OVX model helped me understand the importance of model justification before animal experiments. | □ | □ | □ | □ | □ |
| **F2** | The OVX experiment helped me understand pain assessment and welfare risks during surgical procedures. | □ | □ | □ | □ | □ |
| **F3** | The OVX experiment helped me understand the specific application of anesthesia, analgesia, and postoperative care. | □ | □ | □ | □ | □ |
| **F4** | The OVX experiment helped me master methods for postoperative condition observation and welfare monitoring in animals. | □ | □ | □ | □ | □ |
| **F5** | The OVX experiment helped me understand the ethical requirements for sampling, euthanasia, and confirmation of death. | □ | □ | □ | □ | □ |

**Part III: Overall Evaluation**

| **Item No.** | **Item** | **1** | **2** | **3** | **4** | **5** |
| --- | --- | --- | --- | --- | --- | --- |
| **G1** | This course improved my ability to apply ethical principles to the practice of animal experimentation. | □ | □ | □ | □ | □ |
| **G2** | This course improved my understanding of standardized management of animal experimentation. | □ | □ | □ | □ | □ |
| **G3** | I am satisfied with the overall teaching effectiveness of this course. | □ | □ | □ | □ | □ |
